# Increase in Viral Load in Patients With SARS-CoV-2 Delta Variant Infection in the Republic of Korea

**DOI:** 10.3389/fmicb.2022.819745

**Published:** 2022-03-03

**Authors:** Jeong-Min Kim, Jee Eun Rhee, Myeongsu Yoo, Heui Man Kim, Nam-Joo Lee, Sang Hee Woo, Hye-Jun Jo, Donghyok Kwon, Sangwon Lee, Cheon Kwon Yoo, Eun-Jin Kim

**Affiliations:** ^1^Division of Emerging Infectious Diseases, Bureau of Infectious Disease Diagnosis Control, Korea Disease Control and Prevention Agency, Cheongju-si, South Korea; ^2^Division of Public Health Emergency Response Research, Korea Disease Control and Prevention Agency, Cheongju-si, South Korea; ^3^Korea Disease Control and Prevention Agency, Cheongju-si, South Korea; ^4^Bureau of Infectious Disease Diagnosis Control, Korea Disease Control and Prevention Agency, Cheongju-si, South Korea

**Keywords:** viral load, delta variant, coronavirus disease 2019 (COVID-19), severe acute respiratory syndrome coronavirus 2 (SARS-CoV-2), transmissibility

## Abstract

Severe acute respiratory syndrome coronavirus 2 (SARS-CoV-2) spread rapidly, causing in COVID-19 being declared a global pandemic by the World Health Organization. The key variants include alpha, beta, gamma, and delta; these exhibit high viral transmission, pathogenicity, and immune evasion mechanisms. The delta variant, first confirmed in India, was detected in the majority of COVID-19 patients at the recent wave in the Republic of Korea. Here, the features of the delta variant were compared to the earlier waves, with focus on increased transmissibility. The viral load, from the initial days of infection to 14 days later, was compared based on epidemiological data collected at the time of confirmed diagnosis. The increased viral load observed in the delta variant-led infections influences the scale of the wave, owing to the increased rate of transmission. Infections caused by the delta variant increases the risk of hospitalization within 14 days after symptom onset, and the high viral load correlates with COVID-19 associated morbidity and mortality. Therefore, the future studies should compare the trend of disease severity caused by the high viral load of delta variant with previous waves and analyze the vaccine effects in light of the delta variant of fourth wave.

## Introduction

Severe acute respiratory syndrome coronavirus 2 (SARS-CoV-2), which causes coronavirus disease 2019 (COVID-19), was first detected in China in December 2019. The initial symptoms of COVID-19 were fever, cough, and sore throat, followed by reports of pneumonia with an unidentified cause that developed into severe disease (Huang et al., 2020; Zhou et al., 2020; Zhu et al., 2020). After the rapid spread of SARS-CoV-2, the World Health Organization [WHO] (2020) declared COVID-19 as a global pandemic on January 30, 2020. As of October 25, 2021, the number of confirmed COVID-19 patients worldwide was 243,260,214, with 4,941,039 deaths, giving a mortality rate of 2.03% (WHO). In the Republic of Korea, 354,355 patients tested positive and 2,788 deaths occurred, resulting in a mortality rate of 0.79%. COVID-19 infections surged in the Republic of Korea during the first wave in the first half of 2020, second wave in the second half of 2020, third wave between November 2020 and early 2021, and delta variant-led fourth wave which is still ongoing. Most deaths occurred in patients with comorbidities or in elderly patients with compromised immunity (Cherian et al., 2021; Kwon et al., 2021).

The GISAID (global initiative on sharing all influenza data) categorized SARS-CoV-2 into 10 clades (S, L, V, G, GH, GR, GV, GRY, and GK among others) based on sequencing data. Virus monitoring efforts focus on variants of concern and variants of interest, classified based on the risk that these variants pose to public health (World Health Organization [WHO], 2020). The variants of concern include alpha, beta, gamma, and delta, all of which show distinct features in their viral transmission, pathogenicity, and immune evasion mechanisms. These differences are mainly related to amino acid substitutions in the receptor binding domain (RBD), which facilitates binding of the viral spike (S) protein to host cells (Davies et al., 2021; Faria et al., 2021; Ong et al., 2021a). The delta variant, first detected in India, is prevalent worldwide. This variant belongs to the B.1.617.2, AY.1∼39 lineage of the G clade and mainly harbors a deletion of amino acids 157–158 and L452R, T478K, D614G, and P681R mutations in the gene encoding S protein (Scripps). Compared to the alpha variant, the delta variant is ∼1.6-fold more transmissible and causes ∼2.3-fold more severe disease based on data from inpatients (Public Health England). Among completely vaccinated individuals, the prevention rate of SARS-CoV-2 infection was 88% in those administered the Pfizer vaccine and 60% in those administered the AstraZeneca vaccine based on symptomatic infection at 14 days after complete vaccination (Public Health England). Notably, the signature mutation in the delta variant increased viral transmission and was the major cause of worldwide outbreaks (Kang et al., 2021; Li et al., 2021). Therefore, understanding transmission of the delta variant is critical for controlling and preventing large-scale public health crises.

The delta variant was detected in most patients with COVID-19 during the recent waves. In this study, the features of the delta variant were compared with those causing disease during the first, second, and third waves in 2020 in the Republic of Korea, focusing on its increased transmissibility. The viral loads from the initial day of infection to 14 days later were compared based on epidemiological data collected at the time of confirmed diagnosis. We aimed to generate sufficient evidence to enable an appropriate response to waves caused by the delta variant.

## Materials and Methods

### Specimen Collection and Real-Time RT-PCR for SARS-CoV-2

Overall, 23,940 COVID-19-positive swab specimens (nasopharyngeal and oropharyngeal) were collected, among which 3,934 specimens were from cases reported in the first outbreak, 18,158 specimens were from cases reported in the second outbreak in 2020, and 1,848 specimens were from patients with the delta variant infection in 2021. The specimens were subjected to RNA extraction followed by real-time RT-PCR as described previously. The primer and probe sequences used for RNA-dependent RNA polymerase gene detection were: 5′-GTGARATGGTCATGTGTGGCGG-3′ (Forward), 5′-CARATGTTAAASACACTATT AGCATA-3′ (Reverse) and 5′-CAGGTGGAACCTCATCAGGAGATGC-3′ (Probe in 5-FAM/3′-BHQ format) and the primer and probe sequences used for E gene detection were: 5′-ACAGGTACGTTAATAG TTAATAGCGT-3′ (Forward), 5′-ATATTGCAGCAGTACGC ACACA-3′ (Reverse) and 5′-ACACTAG CCATCCTTACTG CGCTTCG-3′ (Probe in 5-FAM/3′-BHQ format) (Kim et al., 2020). Briefly, RNA was extracted from 140 μL of the sample using a Qiagen viral RNA mini kit according to the manufacturer’s protocol (Hilden, Germany). Real-time RT-PCR was performed using the extracted RNA, and the Ct value of the SARS-CoV-2 target gene was determined. All specimens were handled in a biosafety cabinet according to laboratory biosafety guidelines of the Korea Disease Control and Prevention Agency for COVID-19.

### Virus Full-Genome and Spike Protein Sequencing

For full-genome sequencing, cDNA was amplified from total RNA using ARTIC primer pools.^1^ Libraries were prepared using the Nextera DNA Flex Library Prep Kit (Illumina, San Diego, CA, United States), and sequencing was performed on an MiSeq instrument using a MiSeq reagent kit V2 (Illumina, San Diego, CA, United States) to obtain an average genome coverage of >1,000 × for all samples. The reads were trimmed and mapped to the reference genome Wuhan-Hu-1 (GenBank: MN908947.3) using CLC Genomics Workbench version 20.0.3 (CLC Bio, Aarhus, Denmark) (Park et al., 2021). The lineages and clades of the SARS-CoV-2 sequences were assigned using Nextclade v1.7.1 (Nextstrain Project) and PANGOLIN (Rambaut et al., 2020).

The S protein-encoding gene of SARS-CoV-2 was amplified using one-step RT-PCR (Qiagen, Hilden, Germany) with six primers; three overlapping fragments were amplified. Primers were selected from ARTIC primer pools. The amplified PCR products were purified and sequenced at Biofact Co.

### Calculation of Viral Copy Number

The plasmids carrying the SARS-CoV-2 E gene were used as the positive control. A standard curve was drawn based on the plasmid concentrations and the regression equation (y = −3.5705x + 39.055; y, Ct; x, copy) was obtained by the standard curve. The copy number of the virus present in the samples was calculated using the equation and expressed as logarithmic value of base 10 (log_10_).

### Data Analyses

Statistical analyses were performed using SAS 9.4 (SAS, Inc., Cary, NC, United States). The frequencies and percentages of categorical variables were obtained, and the median and interquartile range were calculated for the Ct values and age. A generalized linear model and logistic regression analysis were used to examine differences in the Ct values for each variable. Statistical significance was set at 0.05 for all cases.

### Ethics Approval Statement

This study was approved by the Institutional Review Board of the Korea Disease Control and Prevention Agency [KDCA] (2020-03-01-P-A) and designated as a service to public health during the outbreak. Therefore, the board waived the requirement for written consent as outlined in the Title Laboratory Respondence to COVID-19.

## Results

### Characterization of Confirmed Cases

After the first patient with COVID-19 was reported in mid-January 2020, the first wave, which was initiated at a church gathering, occurred in the Republic of Korea in February 2020. This wave was dominated by the S, L, and V clades. However, the G, GH, and GR clades were reported and dominated worldwide after April 2020, with an increased mutation frequency in the S protein-encoding gene. These mutations affected virological features such as the viral transmission and disease severity. The second wave started with the GH clade and was prolonged for a long period during the third wave until April 2021. The latest wave caused by the delta variant, which shows high transmissibility, is currently ongoing (Table 1).

**TABLE 1:** Details of different wave periods analyzed in this study and the virus clades dominant during each period.

	First wave	Second and third waves	Fourth wave (delta variant)
Time period	February 9, 2020–May 4, 2020	May 5, 2020–December 31, 2020	April 1, 2021–July 21, 2021
Characteristics (percentage of clade,%)	S, L, V clade mixed (S; 24%, L; 3%, V; 59%)	GH clade dominant (GH; 98%)	Delta variant (G clade) dominant (G; 90%)

We analyzed the following confirmed cases: 3,934 cases within 14 days of symptom onset from the first wave reported in 2020; 18,158 cases from the second and third waves; and 1,848 cases caused by the delta variant from the recent fourth wave. The clinical symptoms ranged from mild symptoms of fever, cough, and sore throat to a lack of symptoms. The highest rate of infection was observed in 20–29-year-old patients (29.2%) in first wave, whereas an even age distribution was observed during the second and third waves, although a higher percentages of older patients (50–59 years old; 18.9% and 60–69 years old, 17.6%) were observed during these waves. During the fourth wave, patients who were 20–29 years old showed the highest rates of infection (26.9%). In addition, the overall COVID-19 incidence was higher in females (53.1%) than males (46.9%), although infection with the delta variant was higher among males (51.9%). The threshold cycle (Ct) distribution of the SARS-CoV-2 strain isolated during the first wave was mostly 25–30 (30.9%), whereas the second and third waves showed similar distributions of Ct values. The Ct values in individuals showing the highest percentage of infection with the delta variant were significantly low at 15–20 (41.2%), which was higher than in the first, second, and third waves (Table 2).

**TABLE 2:** Characteristics of COVID-19 cases during waves 1–3 (February 9–December 31, 2020) and of delta variant cases during wave 4 (April 22–July 21, 2021) in the Republic of Korea.

Characteristics	Total no. (%)	First wave no. (%)	Second and third waves no. (%)	Fourth wave no. (%)	*p*-value

Total	23,940	3,934	18,158	1,848	
Median age, years (IQR)	48 (Q1 = 29, Q3 = 61)	41 (Q1 = 25, Q3 = 57)	50 (Q1 = 32, Q3 = 63)	35.5 (Q1 = 24, Q3 = 50)	
**Age group, years**					<0.0001
0–9	818 (3.4)	50 (1.3)	712 (3.9)	56 (3.0)	
10–19	1,529(6.4)	224 (5.7)	1,136(6.3)	169 (9.1)	
20–29	3,869(16.2)	1,150(29.2)	2,221(12.2)	498 (26.9)	
30–39	3,062(12.8)	477 (12.1)	2,247(12.4)	338 (18.3)	
40–49	3,409(14.2)	555 (14.1)	2,526(13.9)	328 (17.7)	
50–59	4,398(18.4)	662 (16.8)	3,435(18.9)	301 (16.3)	
60–69	3,759(15.7)	442 (11.2)	3,202(17.6)	115 (6.2)	
70–79	1,930(8.1)	236 (6.0)	1,661(9.1)	33 (1.8)	
≥80	1,166(4.9)	138 (3.5)	1,018(5.6)	10 (0.5)	
**Sex**					<0.0001
Female	12,713(53.1)	2,365(60.1)	9,460(52.1)	888 (48.1)	
Male	11,227(46.9)	1,569(39.9)	8,698(47.9)	960 (51.9)	
**Cycle threshold (Ct)**					<0.0001
<15	4,450(18.6)	271 (6.9)	3,542(19.5)	637 (34.5)	
15–20	5,974(25.0)	562 (14.3)	4,651(25.6)	761 (41.2)	
20–25	5,068(21.2)	790 (20.1)	3,960(21.8)	318 (17.2)	
25–30	5,025(21.0)	1,216(30.9)	3,705(20.4)	104 (5.6)	
>30	3,423(14.3)	1,095(27.8)	2,300(12.7)	28 (1.5)	

*IQR, interquartile range.*

### Daily Average Ct and Viral Load After Symptom Onset

**FIGURE 1:**
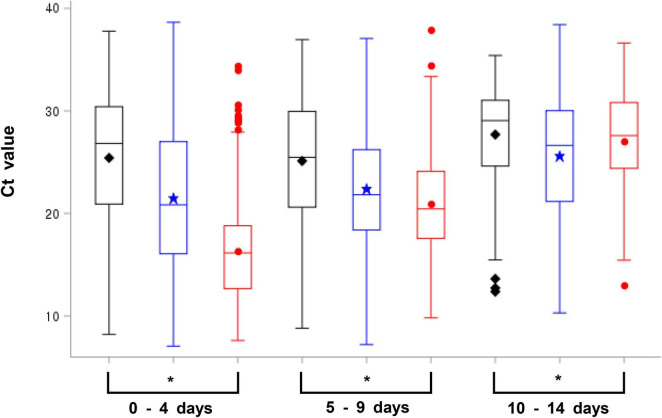
Average cycle threshold (Ct) value of SARS-CoV-2 for each wave on 0–4, 5–9, and 10–14 days following symptom onset [black; first wave, blue: second and third waves, red; fourth wave (delta variant), *p-value < 0.05, Anova and generalized linear model were used to examine differences in the Ct values for each variable].

The total average Ct value within 14 days after symptom onset was 25.47 (range: 8.21–37.78, 95% CI: 33.58) during the first wave, 21.54 (range: 7.06–38.65, 95% CI: 32.5) during the second and third waves, and 16.99 (range: 7.61–37.92, 95% CI: 26.00) during the fourth wave; the Ct value was lower than that during the first wave, the second and third waves. However, the Ct values for each day after the day of symptom onset showed a gradual decrease, with the delta variant Ct on the day of symptom onset exhibiting the maximum lower Ct value than that during the first wave, the second and third waves. Nonetheless, at 10 days after symptom onset, the Ct values showed no significant difference among strains for each wave (Table 3). In addition, the Ct values for the delta variant were lower than those observed during the other waves until day 9 after symptom onset; particularly, the Ct value was significantly lower at 0–4 days after symptom onset (Figure 1). Therefore, the viral load of the delta variant was high soon after symptom onset. In this study, all the Ct values were measured by the same RT-PCR reagent and equipment product based on same primers and probes, but it may have variation because the data has been collected from various confirmatory labs meaning that virus extraction protocol and RT-PCR performance can be different due to lab condition (operator, extraction method, etc.). So, we have conducted a quality evaluation of diagnostic experimental labs to minimize for the potential bias of detection.

**TABLE 3:** Daily average Ct value of COVID-19 cases from the day following onset of symptoms.

	First wave		Second and third waves		Delta variant of fourth wave	
Day after symptom onset	Total case no.	Average Ct (range)	Total case no.	Average Ct (range)	Total case no.	Average Ct (range)
0	2,295	26.14 (8.50–37.14)	11,654	22.08 (7.30–38.65)	519	16.97 (8.25–34.40)
1	314	24.10 (8.21–37.78)	2,199	20.24 (7.40–38.39)	589	15.44 (7.61–30.14)
2	244	23.30 (9.03–37.36)	1,334	19.08 (7.06–37.90)	299	15.84 (8.27–34.00)
3	202	23.37 (8.76–35.29)	872	19.63 (8.01–36.34)	160	16.39 (9.57–29.38)
4	157	23.55 (9.43–34.28)	591	20.67 (8.29–36.09)	80	18.27 (10.69–27.17)
5	148	23.62 (8.80–35.72)	436	21.11 (7.21–37.07)	44	19.55 (9.82–33.36)
6	123	24.72 (12.61–36.37)	296	22.65 (8.66–36.55)	38	19.75 (11.98–34.44)
7	142	26.18 (13.93–36.69)	283	22.78 (9.36–35.06)	26	21.62 (10.41–32.19)
8	86	25.50 (12.51–36.96)	165	23.17 (11.92–35.05)	24	22.62 (12.51–37.92)
9	64	26.47 (13.01–36.62)	99	24.07 (12.13–34.90)	20	22.50 (14.62–29.72)
10	47	27.09 (12.37–34.61)	79	25.25 (10.30–37.53)	9	25.89 (15.44–31.99)
11	27	28.27 (16.00–34.37)	50	26.04 (17.43–38.24)	14	26.71 (17.02–34.92)
12	39	28.11 (15.47–34.84)	32	26.40 (17.14–35.11)	12	28.27 (17.33–36.63)
13	22	27.38 (15.85–34.06)	40	27.85 (19.19–38.42)	7	27.02 (21.77–33.33)
14	24	27.86 (13.63–35.41)	28	26.79 (13.37–35.45)	7	26.99 (13.00–32.72)
Total	3,934	25.47 (8.21–37.78)	18,158	21.54 (7.06–38.65)	1,848	16.99 (7.61–37.92)

**FIGURE 2:**
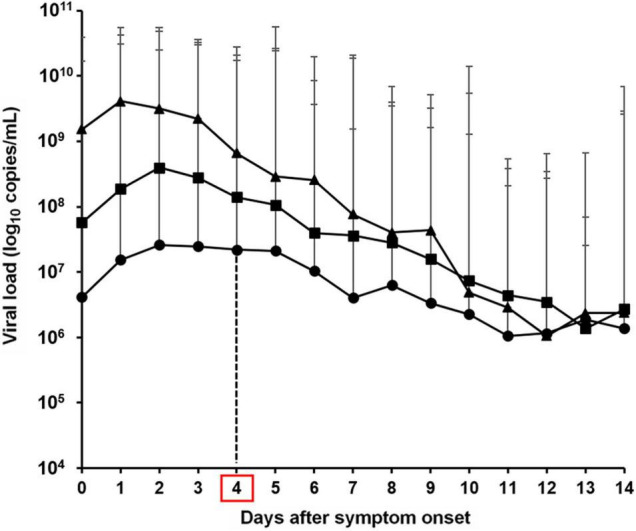
Viral load based on the average cycle threshold (Ct) values of severe acute respiratory syndrome coronavirus 2 from the day following symptom onset in the first–fourth waves in the Republic of Korea [circle; first wave, square; second and third waves, triangle; fourth wave (delta variant)].

Ct values are inversely proportional to the amount of target nucleic acid in a sample. Thus, to quantitatively compare differences based on the viral load, the average Ct within 14 days after symptom onset was converted to the viral load for subsequent analyses. The results showed that patients with delta variant infection had an approximately 237-fold higher viral load (1.51 × 10^9^ log_10_ copies/mL) compared to patients in the first wave (6.38 × 10^6^ log_10_ copies/mL) and 19-fold higher viral load (8.04 × 10^7^ log_10_ copies/mL) compared to patients in the second and third waves. Patients with the delta variant infection during the fourth wave had a viral load of 1.53 × 10^9^ log_10_ copies/mL on the day of symptom onset (day 0), which was approximately 371-fold higher than the viral load of 4.14 × 10^6^ log_10_ copies/mL observed during the first wave and 27-fold higher than the viral load of 5.69 × 10^7^ log_10_ copies/mL observed during the second and third waves. Similarly the viral load on day 4 after symptom onset was approximately 30-fold higher than that during the first wave (6.63 × 10^8^/2.20 × 10^7^ log_10_ copies/mL) and five-fold higher compared to that during the second and third waves (6.63 × 10^8^/1.41 × 10^8^ log_10_ copies/mL). The viral load on day 9 after symptom onset was approximately 13-fold higher compared to that during the first wave (4.33 × 10^7^/3.35 × 10^6^ log_10_ copies/mL) and three-fold higher compared to that during the second and third waves (4.33 × 10^7^/1.57 × 10^7^ log_10_ copies/mL). An overall decreasing trend was observed in all specimens after symptom onset, and the specimens showed no significant difference in viral load after day 10. Notably, the viral load within four days after symptom onset (0–4 days) was markedly higher during the fourth wave, when the delta variant was prevalent, compared to that during the first wave (approximately 30–371-fold), the second and third waves (approximately 5–27-fold), indicating that delta variant caused high viral load at disease onset (Figure 2).

## Discussion

We investigated SARS-CoV-2 viral load as potential factor contributing to the increased in delta variant infections during the fourth wave in 2021 by comparing it with those observed during the first, second, and third wave in the Republic of Korea. In our study, the initial viral load of the delta variant within 4 days after symptom onset was approximately 30–371-fold higher than that during the first wave caused by the S, L, and V clades, including early COVID-19 cases in Wuhan, and was approximately 5–27-fold higher compared to that during the second and third waves, which were mainly caused by the GH clade. Thus, patients with the delta variant infection may show increased viral transmission, which may be an important factor leading to the larger number of COVID-19-positive patients (*n* = 713 per day on average) during the fourth wave compared to that during the first, second, and third waves (*n* = 186 per day on average). A study conducted in India reported that during the first wave, there was a low number of COVID-19-positive cases/million population, but the scenario has changed drastically during the second wave with the delta variant, with over 400,000 confirmed cases/day reported (Sarkar et al., 2021; Tareq et al., 2021). Although current data supports the occurrence of high viral replication and transmission, epidemiological investigations are needed to determine the reproduction number (R) and second attack rate to analyze various characteristics of this variant, including the disease severity.

The RBD of the SARS-CoV-2 spike protein interacts with the host cellular receptor angiotensin-converting enzyme 2 (Bahrami and Ferns, 2020; Deshpande et al., 2021). A mutation in the RBD can directly affect the interaction between the virus and angiotensin-converting enzyme 2. SARS-CoV-2 harboring the D614G mutation, in which glutamic acid (D) at 614 is substituted with glycine (G) on the spike protein, shows a transmission ability. The virus harboring the L452R [lysine (L) substituted at 452 with arginine (R)] mutation shows increased transmission because of its immune evasion ability (Hou et al., 2020; Motozono et al., 2021). In addition, a mutation in the furin cleavage domain at 681–687 prevents proper formation of the S1/S2 unit, thereby altering the viral infection and pathogenicity (Johnson et al., 2021; Lubinski et al., 2021). The delta variant harbors a mutation at 614G on the S protein RBD, as well as the 452R, 478K, and 681R mutations at the furin cleavage site of S protein (Scripps Research; Cherian et al., 2021; Mishra et al., 2021). Increased viral transmission of the delta variant has been attributed to the 614G and 452R mutations, whereas altered viral infection and pathogenicity have been attributed to the 681R mutation.

Our results infer to potential factor that the increased viral load observed in delta variant infections affects the scale of the wave. Additional studies are needed to analyze the correlations between clinical disease severity and the virological features of each wave. Previous studies reported that compared with the alpha variant, infections caused by the delta variant are associated with an increased risk of hospital admission within 14 days of confirmed diagnosis (Ong et al., 2021b; Sheikh et al., 2021), and the high viral load is correlated with COVID-19-associated morbidity and mortality (Fajnzylber et al., 2020; Volz et al., 2021). Therefore, further studies should be performed to compare the trend in disease severity caused by the high viral load of the delta variant with that during previous waves and analyze the vaccine effects considering the delta variant responsible for the fourth wave.

## Data Availability Statement

The SARS-CoV-2 whole genome and Sanger sequences were shared through the GISAID’s EpiCoV (https://www.gisaid.org/). Also, the sequences were deposited by virus names of hCoV-19/South Korea/KCDC, hCoV-19/South Korea/KDCA between accession numbers EPI_ISL_425117 and 6509446.

## Ethics Statement

The studies involving human participants were reviewed and approved by the Institutional Review Board of the Korea Disease Control and Prevention Agency [KDCA] (2020-03-01-P-A) and designated as a service to public health during the outbreak. Therefore, the board waived the requirement for written consent as outlined in the Title Laboratory Respondence to COVID-19. Written informed consent from the participants’ legal guardian/next of kin was not required to participate in this study in accordance with the national legislation and the institutional requirements.

## Author Contributions

J-MK, JR, and E-JK: conceptualization and writing—review and editing. J-MK, JR, MY, and E-JK: methodology. J-MK and MY: software, formal analysis, data curation, and visualization. J-MK and E-JK: validation. J-MK and HK: investigation. SL, CY, and E-JK: resources. J-MK, JR, MY, HK, N-JL, SW, H-JJ, DK, SL, CY, and E-JK: writing—original draft preparation. CY and E-JK: supervision, project administration, and funding acquisition. All authors have read and agreed to the published version of the manuscript.

## Conflict of Interest

The authors declare that the research was conducted in the absence of any commercial or financial relationships that could be construed as a potential conflict of interest.

## Publisher’s Note

All claims expressed in this article are solely those of the authors and do not necessarily represent those of their affiliated organizations, or those of the publisher, the editors and the reviewers. Any product that may be evaluated in this article, or claim that may be made by its manufacturer, is not guaranteed or endorsed by the publisher.

## Footnotes

1
https://artic.network/ncov-2019
